# Use of CytoSorb therapy to treat critically ill coronavirus disease 2019 patients: a case series

**DOI:** 10.1186/s13256-021-03021-y

**Published:** 2021-09-18

**Authors:** Yatin Mehta, Chitra Mehta, Saurabh Nanda, Gaurav Kochar, Joby V. George, Manish Kumar Singh

**Affiliations:** 1 Institute of Critical care and Anesthesia, Medanta The Medicity, Sect 38, Gurgaon, Haryana India; 2 Medanta Institute of Education and Research, Medanta The Medicity, Sect 38, Gurgaon, Haryana India

**Keywords:** CytoSorb, COVID-19, Cytokines, Intensive care unit (ICU), Critical illness

## Abstract

**Background:**

Acute respiratory distress syndrome is an important clinical presentation of respiratory complications caused by severe acute respiratory syndrome coronavirus 2, a novel coronavirus responsible for the ongoing pandemic. The disease is poorly understood, and immunopathogenesis is constantly evolving. Cytokine release syndrome remains central to pathology of coronavirus disease 2019. Antivirals, anticytokine treatment, and other pharmacological approaches have failed to treat it. CytoSorb, an extracorporeal cytokine adsorber that reduces the cytokine storm and other inflammatory mediators in the blood, seems promising in treating severely ill patients with coronavirus disease 2019.

**Case presentation:**

This article presents three cases of Asian ethnicity of severely ill adult patients with coronavirus disease 2019 admitted to intensive care unit who were treated with CytoSorb therapy. All patients used single CytoSorb device. During their clinical course, all patients were prescribed tocilizumab (an interleukin-6 receptor blocker), antivirals, hydroxychloroquine, azithromycin, and other antibiotics and general antipyretic drugs. No vasopressor treatment was required. The patients’ average duration of stay in intensive care unit was 30 days; the average duration of stay in hospital was 31 days. All three patients showed significant improvement in biochemical parameters and clinical outcomes post CytoSorb therapy. C-reactive protein levels decreased by 91.5%, 97.4%, and 55.75 %, and mean arterial pressure improved by 18%, 23%, and 17 % in patient 1, 2, and 3, respectively, on day 7 post-therapy.

**Conclusions:**

All three patients improved clinically and survived.

**Supplementary Information:**

The online version contains supplementary material available at https://doi.org/10.1186/s13256-021-03021-y.

## Background

Coronavirus disease 2019 (COVID-19) has led to the biggest global crisis in recent times. The disease associated with COVID-19 not only has high infectivity and fatality but also resulted in universal economic burden and heavy financial losses [1]. As per the latest (3 June 2021) World Health Organization (WHO) consensus data, 170,812,850 confirmed cases and over 3,557,586 deaths have been reported. In India, approximately 10–20% of patients require intensive care unit (ICU) admission, and 3–10% of patients require intubation and mechanical ventilation [2]. Among the critically ill patients, the mortality rate is 49% [3]. Since its outbreak, concerted efforts are ongoing globally to develop new treatments to overcome the pandemic. Corticosteroids, antivirals, antibiotics, heparin, convalescent plasma, and interleukin-6 (IL-6) receptor blockers are a few possible therapeutic strategies that are being used in treatment of patients with COVID-19 [4].

Pharmacological approaches have shown suboptimal results in critically ill COVID-19 patients. The results of the RECOVERY trial showed that, in the patients hospitalized with COVID-19, dexamethasone (6 mg daily for 10 days) reduced 28-day mortality among those receiving invasive mechanical ventilation or oxygen, but not for the patients who were not receiving respiratory support [5, 6]. On 10 April 2020, the United States Food and Drug Administration (USFDA) granted emergency use authorization (EUA) for emergency use of CytoSorb (M/s CytoSorbents, Inc., Seven Deer Park Drive, Suite K, Monmouth Junction, NJ 08852, USA), to treat patients 18 years of age or older with confirmed COVID-19 admitted to intensive care unit (ICU) with confirmed or imminent respiratory failure [4]. On 6 May 2020, Drug Controller General of India (DCGI) approved emergency use of CytoSorb to reduce proinflammatory cytokines levels in patients with confirmed COVID-19 admitted to the ICU with confirmed or imminent respiratory failure [7]. CytoSorb is an extracorporeal cytokine adsorber designed to broadly reduce cytokine storm and other inflammatory mediators in the blood that could otherwise lead to uncontrolled systemic inflammation, organ failure, and death in many life-threatening illnesses. The device has been safely used worldwide in critically ill patients with COVID-19 [8–11]. Here, we present a case series of three patients who were admitted to ICU at our tertiary care hospital with COVID-19 and were treated with CytoSorb as an adjunct therapy.

## Case presentation

### Patient 1

A 66-year-old woman of Asian ethnicity, with complaints of fever, shortness of breath, and restlessness, was admitted to ICU. She was confirmed to be COVID-19-positive [reverse-transcription polymerase chain reaction (RT-PCR)] with symptoms of pneumonia and associated Guillain–Barre syndrome. Her demographic details and clinical conditions were recorded in case report form (CRF) at the time of admission **(**Table 1). Cytometric bead array (CBA) report revealed high level of interleukin 6 (IL-6), 460 pg/ml. The elevated IL-6, moderate hypoxemia requiring an FiO_2_ of 0.6, and tachypnea (20 breaths/minute) indicated the presence of critical illness. Further, her electroencephalogram (EEG) report suggested the presence of diffuse nonspecific neurophysiological dysfunction. Nerve conduction velocity (NCV) test found the evidence of severe sensorimotor neuropathy affecting all four limbs. Brain magnetic resonance imaging (MRI) showed the presence of multiple small focal and patchy confluent areas of hyperintensity in white matter of both the cerebral hemispheres, though there were no signs of an acute infarct. Findings suggested chronic microvascular ischemic changes and chronic lacunar infarct in the left cerebral hemispheres. Based on the overall picture, CytoSorb therapy was commenced within 24 hours of admission to the ICU.

**Table 1:** Patients’ demographic, clinical characteristics, management, and outcomes

Patient	Sex/age (years)	Comorbidities/indication of CytoSorb	Clinical management	Outcomes
1	F/66	COVID pneumonia Guillain–Barre syndrome Hypertension Diabetes/ Septic with MODS	Hydroxychloroquine Azithromycin Tocilizumab Plasma therapy Other antibiotics Dexamethasone	Discharged
2	M/55	Hypertension Respiratory failure type-1/ Others	Hydroxychloroquine Azithromycin Tocilizumab Plasma therapy Dexamethasone	Discharged
3	M/42	Hypertension Diabetes/ Septic with shock	Dexamethasone Remdesivir Tocilizumab Plasma therapy	Discharged

*F* Female, *M* Male, *COVID* Coronavirus Disease, *MODS* Multiple Organ Dysfunction Syndrome

**Fig. 1.**
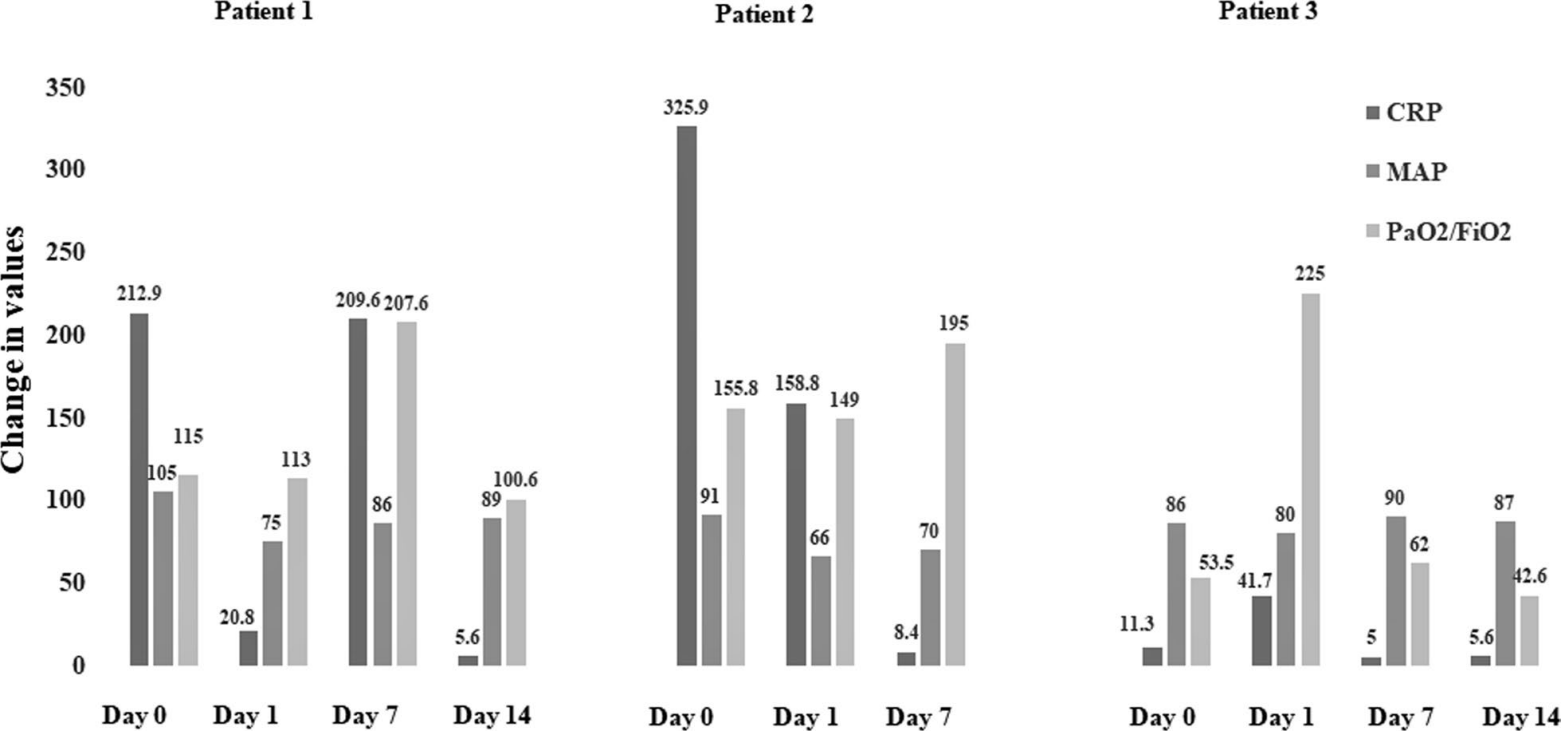
Changes in C-reactive protein, mean arterial pressure, and PaO_2_/FiO_2_ in all patients from day 0 to post-therapy day 1, day 7, and day 14. CRP, C-reactive protein (mg/L); MAP, mean arterial pressure (mmHg); PaO_2_/FiO_2_, ratio of arterial oxygen partial pressure (mmHg) to fractional inspired oxygen (in fraction)

**Fig. 2.**
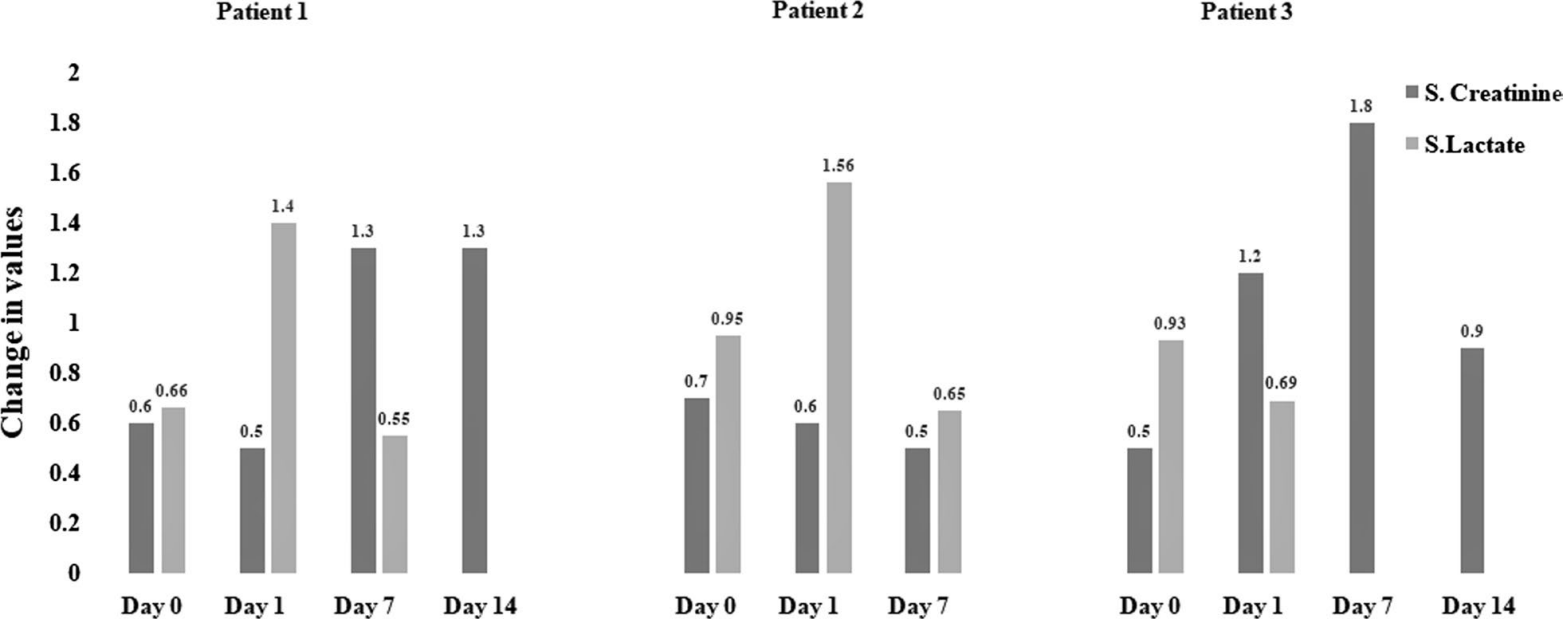
Changes in serum creatinine and lactate in all patients from day 0 to post-therapy day 1, day 7 and day 14. S. Creatinine: serum creatinine (µmol/L), S. Lactate, serum lactate (mmol/L)

Details of CytoSorb treatment and its mechanism are given in Additional file 1. Sustained low-efficiency dialysis (SLED) single CytoSorb device was used for 10 hours with a pump flow rate of 150 ml/minute. By the end of treatment, a sharp decline (> 90%) in C-reactive protein (CRP) values was recorded on the following day (day 1 post-therapy) compared with baseline. There was also a decrease in IL-10 levels (from 12 to 8 pg/ml) after CytoSorb therapy. Other parameters, including mean arterial pressure (MAP), serum lactate, and serum creatinine, continued to improve till end of therapy. At the end of therapy, PaO_2_/ FiO_2_ level also improved. Figures 1 and 2 summarize the change of these during the first 14 days after hemoadsorption therapy. The patient received the recommended therapies of COVID-19 treatment protocol that included injection tocilizumab, plasma therapy, and antibiotics. For Guillain–Barre syndrome, she was prescribed intravenous immunoglobulin (25 g intravenously once daily for 2 days). She was discharged in stable condition after 45 days in ICU.

### Patient 2

A 55-year-old man of Asian ethnicity with hypertension and respiratory failure was admitted to ICU. He tested positive for COVID-19 by RT-PCR. His demographic details and clinical conditions are presented in Table 1. He initially required mechanical ventilation with an infusion of catecholamines. Due to poor response to ventilatory support and progressive hypoxia, (PaO_2_/ FiO_2_: 158 mmHg), CytoSorb therapy was started within 72 hours of admission to ICU and continued for 8 hours, maintaining the flow rate at 150 ml/minute incorporating the CytoSorb device in the SLED circuit. Details of CytoSorb treatment and its mechanism are given in Additional file 1. Post CytoSorb therapy, CRP values decreased on day 1 compared with baseline (51.2%) and day 7 (97.4%). Improvement was also observed in other parameters, which are shown in Figs. 1 and 2 for day 1, day 7, and day 14 post-therapy. The patient was also prescribed tocilizumab (IL-6 blocker) and convalescent plasma therapy. Following the treatment, ventilatory support was discontinued on day 6, and the patient showed clinical recovery in 11 days. On discharge, his condition was stable. His oxygen saturation level was maintained at 96%. Overall, 25% improvement in PaO_2_/FiO_2_ level was noted.

### Patient 3

A 42-year-old man of Asian ethnicity, known case of COVID-19 pneumonia, was admitted to the hospital. The patient had complaints of cough and fever for the last 4–5 days. His demographic details and clinical conditions were recorded in CRF at the time of admission (Table 1). At the time of admission, his SpO_2_ level was 92% on nonrebreather mask (15 L/minute). MRI brain showed no evidence of acute hypoxic-ischemic or acute metabolic encephalopathy. Chest X-ray showed right pleural effusion and multiple hazy opacities in bilateral lung fields. Right lung was comparatively more affected, showing total collapse and associated mild pleural effusion with interlobular septal thickening. He was admitted to ICU and was immediately started with a single CytoSorb device maintaining the flow rate at 110 ml/minute with HD for 7.5 hours. Details of CytoSorb treatment and its mechanism are given in Additional file 1. Along with CytoSorb therapy, the patient was also given noninvasive mechanical ventilation support. Since he stayed hypoxemic, he was intubated and put on invasive mechanical ventilation. He required vasopressor support for hemodynamic instability, and also underwent percutaneous tracheostomy for prolonged ventilator need. During treatment, he developed polymicrobial sepsis and acute psychosis, which resolved by the end of drug therapy. Laboratory parameters were recorded on day 1, till day 14 post-therapy. IL-6 levels from baseline decreased by 24.5% (from 2256 to 2808 pg/ml), 65% (from 2256 to 776 pg/ml), and 30% (from 2256 to 1572 pg/ml) on day 1, day 7, and day 14 post-therapy, respectively. IL-10 levels also decreased from baseline by 37.5% (from 16 to 10 pg/ml) and 25% (16 to 12 pg/ml) day 7 and day 14 post-therapy, respectively. By the end of treatment, a sharp decline in CRP values, 56%, was recorded. Improvement was also reported in serum lactate values: 25% on day 1 post-therapy. An improvement was also observed in other parameters as shown in Figs. 1 and 2 for day 1, day 7 and day 14 post-therapy. Drug therapy was prescribed as per the COVID-19 protocol. He was administered remdesivir, tocilizumab, and plasma therapy. The patient was kept in ICU for 34 days. At discharge, his condition was stable.

## Discussion

In this case series, patients suffering from critical illness of different severities due to COVID-19 were treated with CytoSorb therapy. We aimed to observe the extent of improvement in laboratory and clinical parameters after hemoadsorption.

Before starting the therapy, their clinical conditions were matched with the criteria suggested as per the guidelines recommended by global COVID-19 associations/societies to treat critically ill COVID-19 patients [2, 12, 13]. They were admitted to ICU on the basis of their severity of acute respiratory distress syndrome (ARDS) and levels of biochemical parameters as defined by clinical guidelines. As per the position statement of the Indian Society of Critical Care Medicine, ICU admission is recommended in cases of PaO_2_/FiO_2_ < 300, tachypnea ≥ 30 breaths/minute, or oxygen saturation ≤ 94%. Respiratory failure, multiple organ dysfunction (MODS), and septic shock are considered as life-threatening conditions [2]. All patients in this case series were admitted to hospital with PaO_2_/FiO_2_ level < 150 and respiratory rate (RR) > 25 breaths/minute, clear indicators for admission in ICU. Among these, patient 2 had severe respiratory failure at the time of admission.

Critically ill COVID -19 patients often present with some sort of hyperinflammation (also called cytokine storm in the most severe cases) due to the dysregulated inflammatory response due to infection with excessive activation of immune cells and generation of proinflammatory cytokines; IL-6, IL-10, tumor necrosis factor (TNF), and others. Indeed, in critically ill patients with COVID-19, it is a frequently reported phenomenon [14, 15]. Our results are also in accord with this, indicating some sort of an increased proinflammatory hyperactivity based on elevated CRP and IL-6 values.

Our results are also consistent with a recently published prospective randomized proof of concept pilot study in patients in septic shock published by Hawchar *et al.* [16], and with another study in which a patient with COVID-19 interstitial pneumonia was treated with the combination of tocilizumab and hemoadsorption [17].

Use of anticytokine therapies, such as tocilizumab (anti-IL-6) has been suggested by some studies as a promising treatment, which may be capable of reducing the risk of invasive mechanical ventilation and death in patients with severe COVID-19 [17–19]. According to a recent recommendation from Italy (Brescia Renal COVID Task Force of using CytoSorb in COVID-19 patients), the therapy should be started within the first 6 to maximum 24 hours after the start of standard therapy and should be continued for 24–48 hours after initiating tocilizumab treatment. As CytoSorb adsorbs molecules of 5–50 kDa, tocilizumab (148 kDa) is not removed by this therapy owing to its larger size. Therefore, it can be used along with CytoSorb therapy. In this case series, all patients were started on CytoSorb therapy within 72 hours of their admission to ICU depending on their critical level of illness and continued for 24 hours (Additional file 1). It has been reported that starting the therapy within 24–48 hours of a sepsis diagnosis could lead to decreased mortality in both medical and post-surgical patients [20–22]. It is also acknowledged that, in some cases, the use of more than one adsorber may be necessary, given the individual patient’s response to therapy.

All three patients showed speedy recovery as the period of ICU stay was comparatively shorter. Similar findings are also reported in a systemic review conducted by Rees *et al*., which included 52 studies from inside and outside China to determine the length of stay (LOS) of patients with COVID-19 in hospital and in ICU. The study reported median duration of 5–29 days of hospitalization. For critically ill patients, LOS was reported for more than 50 days [23]. In this case series, all patients were discharged from ICU within 11–45 days in clinically stable condition. An improvement in other vital parameters like heart rate (HR), RR, blood pressure (BP), SpO_2_, and body temperature was also reported.

Generally, patients with septic shock and sepsis are prescribed vasopressors or inotropes in order to maintain hemodynamic parameters [24].

All three patients in our study had a history of hypertension. Two patients (1 and 3) had diabetes with septic shock and sepsis with MODS, respectively. Their blood pressure values were normal at the time of discharge (patient 1, 126/60 mmHg; patient 2, 120/59 mmHg; patient 3, 149/73 mmHg). This indicates that CytoSorb therapy helped to wean them from vasopressor support, hence improving their hemodynamic stability. For all three patients, clinical management included antibiotics; hydroxychloroquine, azithromycin, remdesivir, tocilizumab, convalescent plasma therapy, and general antipyretic drugs (Table 1).

To date, the device has been used widely in more than 3000 critically ill patients infected with COVID-19 in 30 countries [25]. Various clinical trials are ongoing globally to investigate the effects of CytoSorb in COVID-19 patients [8]. In general, it has been used safely in more than 100,000 treatments worldwide, primarily in the treatment of systemic hyperinflammation in a wide variety of life-threatening conditions such as septic shock [26, 27], influenza, ARDS, secondary hemophagocytic lymphohistiocytosis (HLH), trauma, liver failure, pancreatitis, and multiple organ failure [25, 28]. However, the use of CytoSorb is globally accepted by clinicians and researchers only to be employed as an adjunctive therapy to lower cytokine storm, but not as a primary intervention to remove the virus [8, 17, 29].

## Conclusion

Extracorporeal cytokine removal could be a useful adjuvant therapy to overcome hyperinflammation in critically ill patients. It could also be considered as a potential therapeutic option to manage the serious complications of hyperinflammation and cytokine release syndrome in critically ill COVID-19-infected patients. However, patients undergoing

CytoSorb therapy should be carefully monitored with drug levels (when possible), and supplemented with additional doses as needed.The encouraging results of our case series indicate its potential in COVID-19 patients, and suggest a prospective study to further emphasize its importance in managing conditions associated with exaggerated inflammatory response.

## Supplementary Information

**Additional file 1.** Additional figure and table.

## Data Availability

The data were retrieved from medical files of the patients and case report forms. These are available from Dr. Yatin Mehta, the corresponding author, upon reasonable request.
