# Supplementary material for: Use of CytoSorb therapy to treat critically ill coronavirus disease 2019 patients: a case series
Source: J Med Case Rep. 2021 Sep 18;15:476. doi: 10.1186/s13256-021-03021-y (PMC8448661; doi:10.1186/s13256-021-03021-y)
Supplement: Supplementary file 1 — Additional file 1. Additional figure and table. [file 13256_2021_3021_MOESM1_ESM.docx]

**CytoSorb^®^**  **Treatment: Events in Patients**

| **Patient 1**  **(66Y/F)** | **Mechanical Ventilation Start Date**  Day 1 Post hospitalization | **Mechanical Ventilation End Date**  Discontinued Day 5 | **CytoSorb^®^**  **Start Date**  Day 3 Post hospitalization | **Type of Dialysis and**  **Flow Rate**  SLED  150 ml/min | **CytoSorb^®^**  **End Date**  After 24 hrs:  Day 4 Post hospitalization | **Follow up**  Till Day 14 post hospitalization | **Date of Discharge**  Post 45 days of hospitalization |
| --- | --- | --- | --- | --- | --- | --- | --- |
| **Patient 2**  **(55Y/M)** | **Mechanical Ventilation**  Day 1 Post hospitalization | **Mechanical Ventilation End Date**  Discontinued Day 6 | **CytoSorb^®^** **Start Date**  Day 3 Post hospitalization | **Type of Dialysis and**  **Flow Rate**  150 ml/min with SLED | **CytoSorb^®^**  **End Date**  After 24 hrs:  Day 4 Post hospitalization | **Follow up**  Till Day 7 post hospitalization | **Date of Discharge**  Post 11 days of hospitalization |
| **Patient 3**  **(42Y/M)** | **Mechanical Ventilation**  Day 1 Post hospitalization | **Mechanical Ventilation End Date**  Discontinued same day | **CytoSorb^®^ Start Date**  Day 2 Post hospitalization | **Type of Dialysis and**  **Flow Rate**  110 ml/min HD for 7.5 hours | **CytoSorb^®^** **End Date**  After 24 hrs:  Day 3 Post hospitalization | **Follow up**  Till Day 7 post hospitalization | **Date of Discharge**  Post 34 days of hospitalization |

**CytoSorb Mechanism and Use**

The CS device was approved in Europe in 2011 and can be used as a stand-alone therapy or in combination with extracorporeal circuits, both in pre-dialyzer and post-dialyzer mode **(Fig. 1).**

**Fig. 1.** CytoSorb circuit: (**a**) as a stand-alone therapy, (**b**) in the pre-dialyzer mode, (**c**) in the post-dialyzer mode (Source: Cyto-Sorbents Europe GmbH).


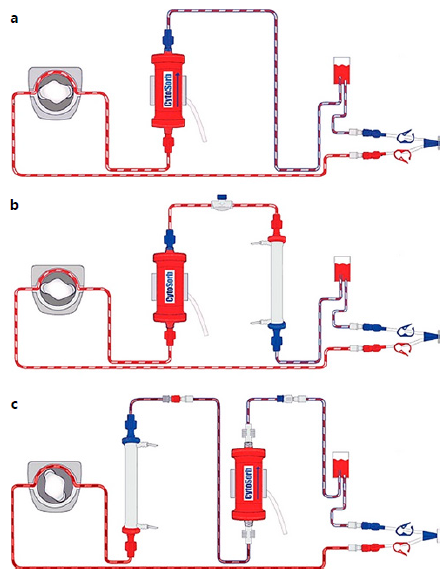


CS is made up of biocompatible, highly porous, polyvinyl-pyrrolidone-coated polystyrene-divinyl-benzene polymer bead. Its estimated size is 300–800 μm with a total surface area of more than 40,000 m2. The large surface area gives greater capacity for clearance than available dialyzers. Substances removal from whole blood is based on pore capture and surface adsorption. CS use is compatible with both systemic heparin and regional citrate anti-coagulation. The typical duration of therapy is up to 24 h per session, daily for 2–7 consecutive days. Blood flow is set in the range of 150–700 mL/min. CS use is indicated in clinical conditions with cytokines elevation. It is effective in targeting middle-molecular-weight uremic and low-molecular-weight toxins with a wide range of molecular weights (∼5–60 kDa). It however, does not capture endotoxins and interleukin (IL)-10, as generally adsorption is effective in pore size-dependent manner

[***Reference: Ankawi G, Xie Y, Yang B, Xie Y, Xie P, Ronco C. What have we learned about the use of cytosorb adsorption columns?. Blood purification. 2019;48(3):196-202.***](https://www.karger.com/Article/PDF/500013)
